# Role of the Hypoxia-inducible factor-1 alpha induced autophagy in the conversion of non-stem pancreatic cancer cells into CD133^+^ pancreatic cancer stem-like cells

**DOI:** 10.1186/1475-2867-13-119

**Published:** 2013-12-05

**Authors:** Haitao Zhu, Dongqing Wang, Yanfang Liu, Zhaoliang Su, Lirong Zhang, Fangfang Chen, Yuepeng Zhou, Yingying Wu, Ming Yu, Zhijian Zhang, Genbao Shao

**Affiliations:** 1 The Affiliated Hospital of Jiangsu University, Zhenjiang 212001, China; 2 Department of Biology, Jiangsu University, Zhenjiang 212013, China; 3 The First People’s Hospital of Zhenjiang, Zhenjiang 212001, China; 4 Department of Radiology, The Affiliated Hospital of Jiangsu University, Jiangsu University, 438 Jiefang Road, 212013, Zhenjiang, Jiangsu Province, China; 5 Department of Biology, School of Medical Science and Laboratory Medicine, Jiangsu University, 301 Xuefu Road, 212013, Zhenjiang, Jiangsu Province, China

**Keywords:** Pancreatic cancer stem cell, HIF-1α, Autophagy

## Abstract

The initiation and progression of various solid tumors, including pancreatic carcinoma, are driven by a population of cells with stem cell properties, namely cancer stem cells (CSCs). Like their normal counterparts, CSCs are also believed to rely on their own microenvironment termed niches to sustain the population. Hypoxia-inducible factor-1α (HIF-1α) is a major actor in the cell survival response to hypoxia. Recently, several researchers proposed that non-stem cancer cells can convert to stem-like cells to maintain equilibrium. The present study focuses on whether non-stem pancreatic cancer cells can convert to stem-like cells and the role of HIF-1α and autophagy in modulating this conversation. The non-stem pancreatic cancer cells and pancreatic cancer stem-like cells were separated by magnetic sorting column. Intermittent hypoxia enhanced stem-like properties of non-stem pancreatic cancer cells and stimulated the levels of HIF-1α, LC3-II and Beclin. Enhanced autophagy was associated with the elevated level of HIF-1α. The conversation of non-stem pancreatic cancer cells into pancreatic cancer stem-like cells was induced by HIF-1α and autophagy. This novel finding may indicate the specific role of HIF-1α and autophagy in promoting the dynamic equilibrium between CSCs and non-CSCs. Also, it emphasizes the importance of developing therapeutic strategies targeting cancer stem cells as well as the microenvironmental influence on the tumor.

## Introduction

Pancreatic cancer is the fourth leading cause of cancer-related death in the United States. It is characterized by rapid progression, early metastasis, and a limited response to chemotherapy [1]. Despite advances in our understanding of the molecular biology of pancreatic cancer, the outcome of systemic treatment for this disease remains unsatisfactory. Inhibition of tumour growth and metastasis by inhibitors of matrix metalloproteinases and angiogenesis had little effect on the natural progress of this malignancy and did not produce dramatic improvements in patient survival rate [2,3]. More understanding of molecular mechanisms of cell growth and proliferation is urgently needed.

A distinct population of cancer cells, namely cancer stem cells, had been defined and characterized in several human cancers, including pancreatic cancer [4-6]. Most described CSCs were isolated from solid tumors based on the expression of surface markers [7,8]. CD133 is a marker protein typically used to identify and isolate human pancreatic CSCs [9]. The properties that distinguish CSCs from the rest of the tumor cells are their ability to self-renew, differentiate into heterogeneous types of tumor cells, and sustain tumor growth in *vivo*[10]. In this study, we designated CD133 positive cells as pancreatic cancer stem-like cells whereas CD133 negative cells utilized as non-stem pancreatic cancer cells. Due to failing to eradicate the CSCs population, traditional cancer therapies are effective at reducing tumor mass but often fail to produce long-term clinical complete remissions. A number of studies in recent years have demonstrated that CSCs are located in specialized microenvironments within tumor [11-13].

Most solid tumors are characterized by hypoxic areas originating from an imbalance between oxygen supply and expenditure in the actively proliferating tumor cells. It is becoming increasingly clear that the intrinsic properties of CSCs are tightly regulated by specific hypoxic microenvironments. Interestingly, several researchers proposed that differentiated cancer cells (non-stem cancer cells) can convert to stem-like cells to maintain equilibrium [14-16]. However, the exact mechanisms of how hypoxic niches induce non-CSCs into CSCs remain largely unknown.

In this study, we first examined the role of intermittent hypoxia in converting the non-stem pancreatic cancer cells into pancreatic cancer stem-like cells. Then, we examined the effective of intermittent hypoxia in changing expression levels of HIF-1α, LC3-II and Beclin. Finally, we tested the relation among HIF-1α, autophagy and the conversation of non-stem pancreatic cancer cells into pancreatic CSCs. Our data showed that conversation of non-stem pancreatic cancer cells into pancreatic CSCs resulted from the elevated HIF-1α and activated autophagy.

## Materials and methods

### Isolated and culture of pancreatic cancer stem cells and non-stem pancreatic cancer cells

The pancreatic cancer cell lines (Panc-1 and BxPC-3, purchased from Cell Bank of China Academy of Sciences, Shanghai, China) were cultured in DMEM-F12 (Gibco, USA) supplemented with 10% FBS (Gibco, USA), 100 U/mL penicillin and 100 U/mL streptomycin, in a humidified atmosphere of 95% air with 5% CO_2_ at 37°C. Cells were passaged with 0.25% trypsin/EDTA every 3 days. Several passage later, cells were seeded and cultured in serum-free medium consisting of DMEM-F12 medium supplemented with 20 ng/mL epidermal growth factor (Peprotech, Rocky Hill, NJ, USA), 20 ng/mL basic fibroblast growth factor (Peprotech, USA), B27 (Invitrogen Life Technologies, Carlsbad, CA, USA), 5 μg/mL insulin, and 2.75 μg/mL transferrin (Sigma-Aldrich, St. Louis, MO, USA) for eight hours to recover surface antigens. Then cells were separated by magnetic sorting column using microbead-conjugated CD133 antibodies (Miltenyi Biotec Ltd., Surrey, UK). CD133 positive cells were designated as pancreatic cancer stem cells whereas CD133 negative cells utilized as non-stem pancreatic cancer cells.

Non-stem pancreatic cancer cells were cultured overnight in DMEM-F12 and 5% serum to allow cell attachment and survival. Then, the medium was removed and the cells cultured in serum-free special medium in order to perform the experiments in identical.

### Intermittent hypoxia environmental exposure

Isolated non-stem pancreatic cancer cells were exposed to 5 cycles of hypoxia and normoxia. Each cycle consisted of a period of 12 h in hypoxia followed by 12 h recovery under normoxia conditions. The medium was changed during the reoxygenation period. For hypoxia induction, cells were cultured in hypoxia chambers (Sanyo,containing 1% O2, 5% CO2, 94% N2.). Nitrogen gas was supplied to the chambers to induce a controlled reduced percentage of oxygen. For normoxia, cells were cultured in incubators containing 5% CO_2_ and approximately 20% O_2_.

### siRNA knockdown of HIF-1αgene and Chemical treatment

Isolated non-stem pancreatic cancer cells were seeded in 12-well plates. When the cell density reached 50% confluence, the cells were transfected with either 40 nmol/L a control siRNA that did not specifically target any gene or HIF-1α-specific siRNA (Suzhou Ribo Life Science CO., Ltd). Transfections were carried out according to the manufacturer’s instructions. Then the cells were put in intermittent hypoxia and normoxia conditions, respectively. Forty-eight hours later, the cells were treated with 30 μL of 3-MA at the concentration of 10 mM and continually cultured for 24 h. The treated cells were then subjected to further experiments.

### Western blot analysis

Cells lysates were subjected to sodium dodecyl sulfate-polyacrylamide gel electrophoresis and transferred to polyvinylidene fluoride membranes (Merck Millipore, USA). Membranes were blocked with 5% (w/v) bovine serum albumin (BSA) in TBST for 1 h at room temperature and incubated overnight with primary antibodies at 4°C. They were subsequently incubated with horseradish peroxidase-conjugated second antibodies. The immunoreactive bands were detected by chemiluminescence (ECL Plus, Merck Millipore) and relevant blots were quantified by densitometry using LANE-1D software. For immune detection, the primary antibody preparations were used as follows: rabbit-anti-human-Oct-4, rabbit-anti-human-c-MYC, rabbit-anti-human-HIF-1α, rabbit-anti-human-LC3B, rabbit-anti-human-Beclin, rabbit-anti-human-β-actin. All antibodies were obtained from Cell Signaling Technology, Inc. (Boston, USA). The secondary antibody preparations either anti-rabbit or anti-mouse were purchased from Boster biotechnology company (Wuhan, China).

### Real-time PCR

Real-time quantitative PCR was carried out with SYBR Green qPCR SuperMix (Bio-Rad) using the CFX-96 system (Bio-Rad). Total cellular RNA was isolated using TRIzol reagent, and cDNA was synthesized from 1 μg of total RNA using oligo dT and murine Moloney leukemia virus reverse transcriptase (Toyobo, Japan). Relative expression levels of the genes were calculated using the 2^-ΔΔCT^ method.

### Flow cytometry analysis

To quantify the converting cells of in non-stem pancreatic cancer cells under intermittent hypoxia with or without siRNA, we measured the expression of the stem related molecular marker CD133 using anti-CD133- PE (Miltenyi Biotec Ltd., Surrey, UK). Cells were harvested, disaggregated to a single cell suspension, and stained as described previously [17].

### Statistical analysis

The significances of differences between groups were analyzed using Student’s t tests. Values of *p* < 0.05 were considered to be significant. All experiments were performed at least in triplicate.

## Results

### Characterization of CSC-like cells

**Figure 1:**
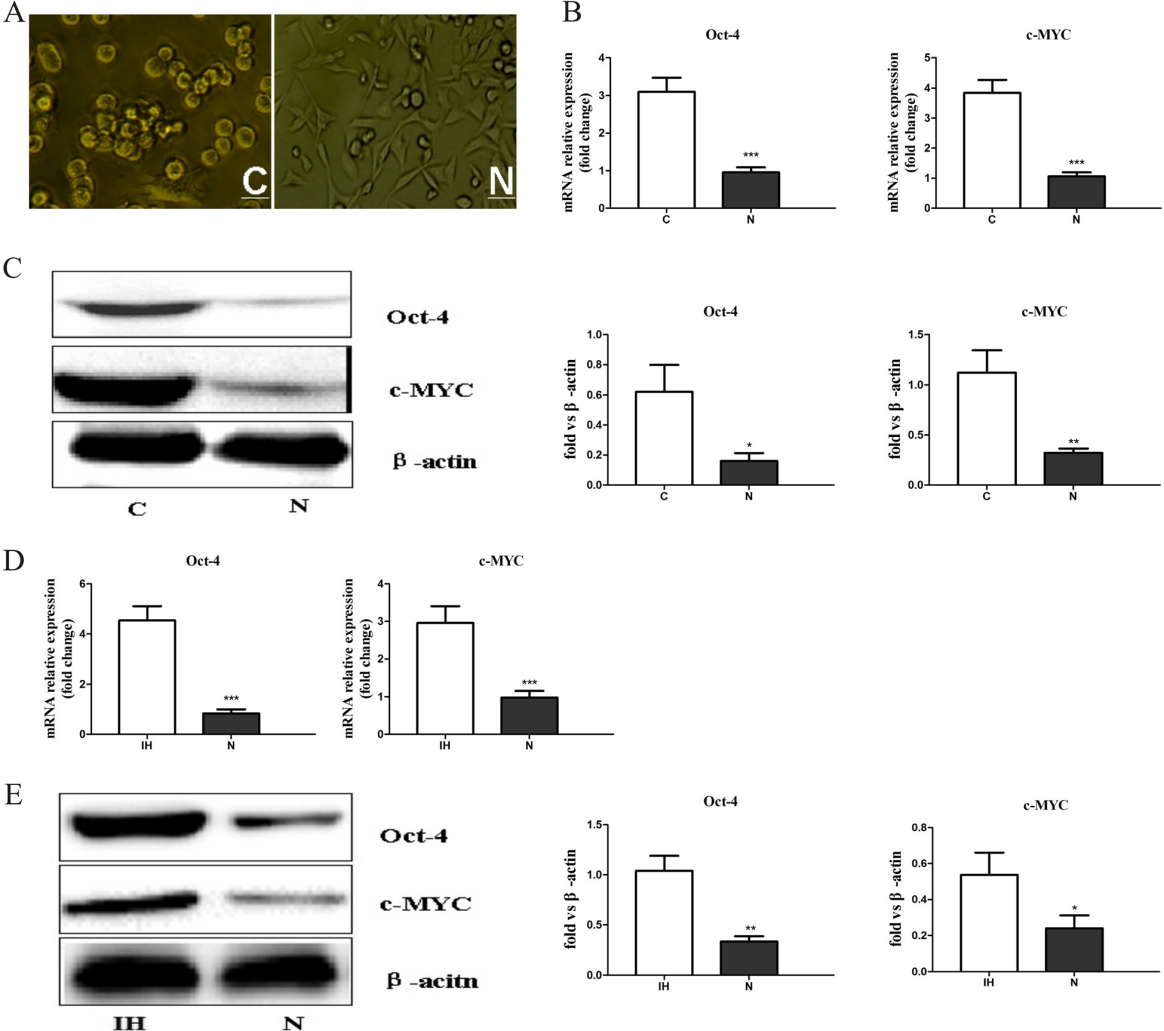
**Characterization of morphology and stemness-related markers between pancreatic cancer stem-like cells and non-stem pancreatic cancer cells. (A)** Phase-contrast images of the pancreatic cancer stem-like cells displayed epithelial-like appearance, while non-stem pancreatic cancer cells displayed fibroblast-like appearance. **(B)** Oct-4, c-MYC genes expression in pancreatic cancer stem-like cells and non-stem pancreatic cancer cells were detected by RT-PCR. **(C)** Expression of Oct-4, c-MYC proteins in pancreatic cancer stem-like cells and non-stem pancreatic cancer cells were detected by Western blotting. Data were normalized to β-actin levels. Experiments were repeated three times with similar data(* *P* < 0.05, **P < 0.01, ***P < 0.001). C represents for pancreatic cancer stem-like cells, N represents for non-stem pancreatic cancer cells. Scale bar equal 50 μm. **(D)** Oct-4, c-MYC gene expression of the bulk pancreatic cells under the intermittent hypoxia (IH) and normoxia (N) conditions were detected by RT-PCR. **(E)** Expression of Oct-4, c-MYC proteins in the bulk pancreatic cells under the intermittent hypoxia (IH) and normoxia (N) conditions were detected by Western blotting. Data were normalized to β-actin levels. Experiments were repeated three times with similar data (* *P* < 0.05, **P < 0.01, ***P < 0.001).

The pancreatic cancer stem-like cells were isolated from the pancreatic cancer cells and their characteristics were examined. It was easily found that the morphology of pancreatic cancer stem-like cells displayed epithelial-like appearance whereas the non-stem pancreatic cancer cells displayed fibroblast-like appearance (Figure 1A). Furthermore, the mRNA and protein levels of Oct-4 and c-MYC were significantly increased in CD133^+^ pancreatic cancer stem-like cells compared to CD133^-^ non-stem pancreatic cancer cells (Figure 1B and C).

### Effective of intermittent hypoxia on maintaining the stem cell properties of bulk pancreatic cells

It has been reported that hypoxia exposure alone can increase the stem-like cell population. At first, we determine whether intermittent hypoxia increase the expression of stem cell associated proteins in the bulk pancreatic cells which referred to the total cells without sorting by the magnetic sorting column using RT-PCR and Western-blot assay. The results demonstrated that the bulk pancreatic cells in intermittent hypoxia displayed an increase in transcript levels of stem cell markers Oct-4 and c-MYC in comparison with the cells cultured in normoxia (Figure 1D). Further, Western-blot had confirmed the up-regulation of Oct-4 and c-MYC expression in intermittent hypoxia conditioned tumor cells (Figure 1E). Thus, our data confirms previous studies showing that hypoxia induces stem cell associated markers including c-myc and Oct4.

### Role of intermittent hypoxia in reprogramming non-stem cancer cells towards a stem-like phenotype

**Figure 2:**
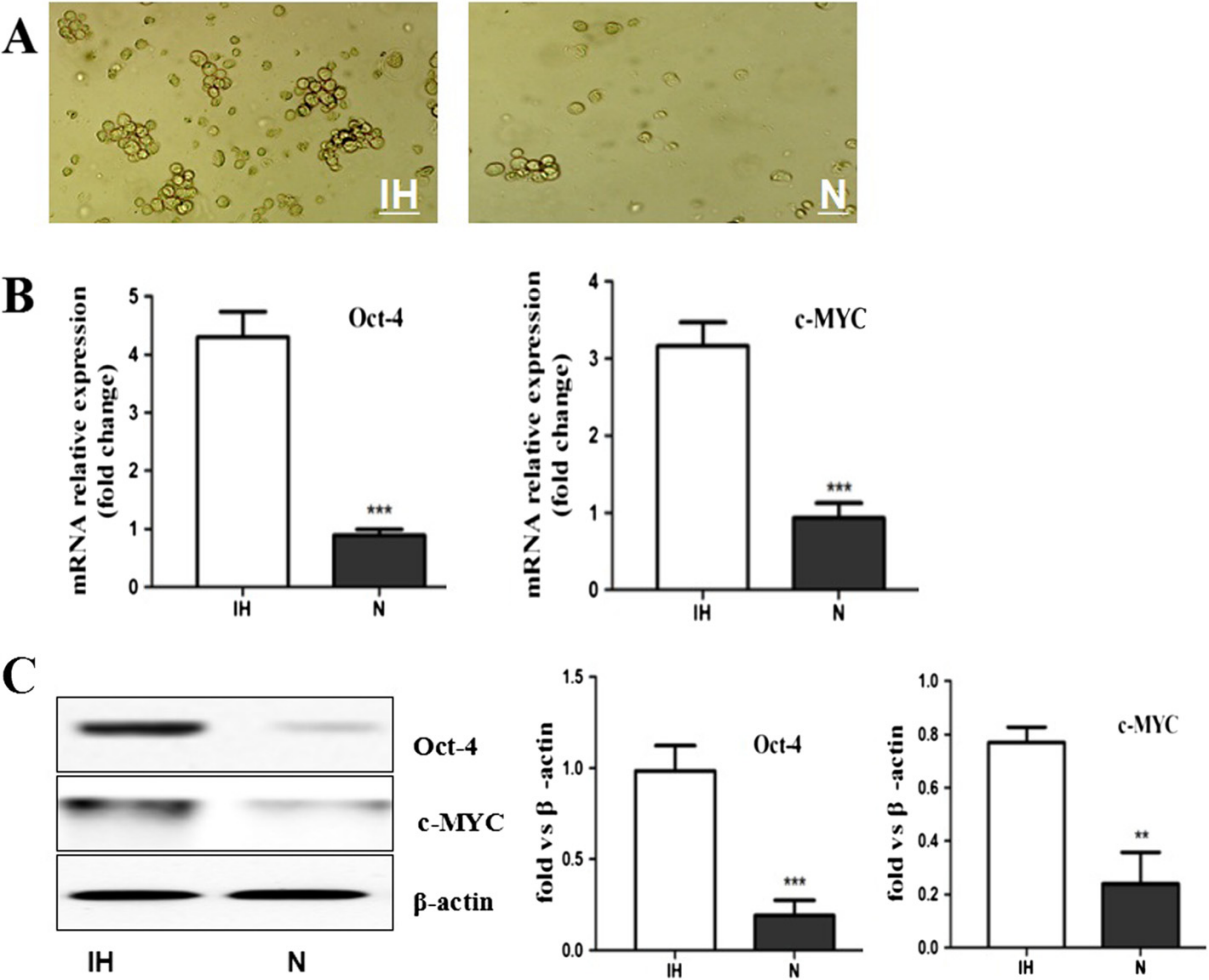
**Intermittent hypoxia reprogrammed non-stem pancreatic cancer cells towards a Stem-like Phenotype. (A)** Phase-contrast images of non-stem pancreatic cancer cells cultured under intermittent hypoxia (IH) and normoxia (N) condition. In intermittent hypoxia condition spheres can be detected while the rate of control cells in normoxia was few. **(B)** Oct-4, c-MYC gene expression of non-stem pancreatic cancer cells under the intermittent hypoxia (IH) and normoxia (N) conditions were detected by RT-PCR. **(C)** Expression of Oct-4, c-MYC proteins in non-stem pancreatic cancer cells under the intermittent hypoxia (IH) and normoxia (N) conditions were detected by Western blotting. Data were normalized to β-actin levels. Experiments were repeated three times with similar data (**P < 0.01, ***P < 0.001).

Conventionally, sphere formation assay is thought to be the method of measuring self-renewal in *vitro*. In the study, the isolated non-stem pancreatic cancer cells were seeded into low adhesion culture plates and treated in intermittent hypoxia. We found that non-stem pancreatic cancer cells cultured under intermittent hypoxia were able to form spheres (Figure 2A). The non-stem pancreatic cancer cells under intermittent hypoxia for 72 h significantly increased the expression levels of Oct-4 and c-MYC in comparison to those under normoxia (Figure 2B and C). These results indicated that intermittent hypoxic microenvironment not only plays a critical role in promoting and maintaining the ability of stem-like cells to self-renew and stemness but also can confer this capability to the non stem-like population.

### Up-regulation of HIF-1α, LC3-II and Beclin express level in non-stem cancer cells under intermittent hypoxia condition

**Figure 3:**
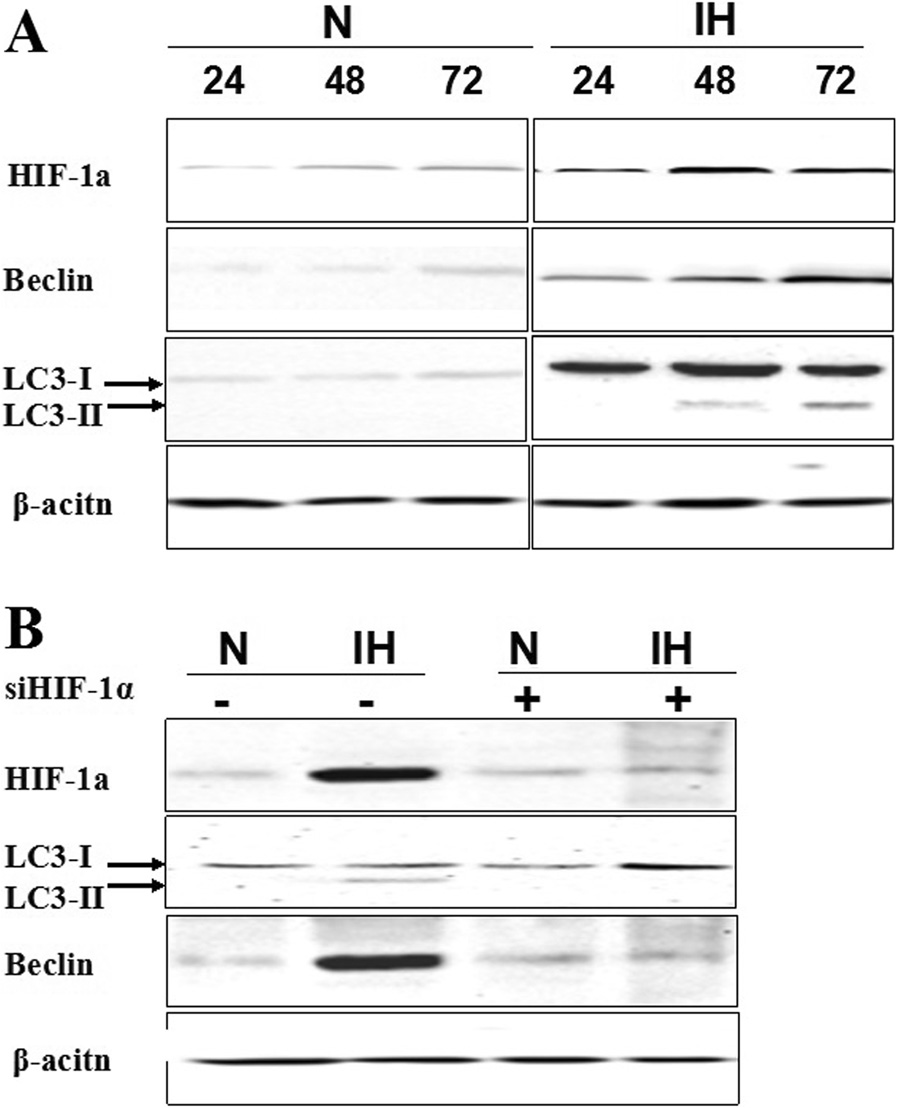
**Enhanced HIF-11α and autophagy related proteins express level in non-stem cancer cells under intermittent hypoxia condition. (A)** Isolated non-stem cancer cells were treated with intermittent hypoxia (IH) and normoxia (N) for the indicated times (24 h, 48 h and 72 h). Expression of HIF-1α, LC3-II and Beclin proteins in non-stem pancreatic cancer cells under the intermittent hypoxia (IH) and normoxia (N) conditions were detected by Western blotting. Data were normalized to β-actin levels. Experiments were repeated three times with similar data. **(B)** Blockade of HIF-1α with siRNA inhabited autophagy in intermittent hypoxia. Expression of HIF-1α, LC3-II and Beclin proteins in non-stem pancreatic cancer cells under the intermittent hypoxia (IH) and normoxic (N) conditions were detected after transfections by Western blotting.

Non-stem pancreatic cancer cells were subjected to intermittent hypoxic conditions for various time periods. Western-blot assay was employed to assess the role of intermittent hypoxia in changing the expression level of HIF-1α, LC3-II and Beclin. Results revealed that the protein levels of HIF-1α, LC3-II and Beclin were significantly increased in a time-dependent manner. Compared to normoxic condition, in intermittent hypoxia, the expression levels of HIF-1α, LC3-II and Beclin were slightly increased in the first 24 h. HIF-1α exhibited the highest levels after 48 h but LC3-II and Beclin did not reach the maximally levels until 72 h (Figure 3A). Meanwhile, cell death rate did not exceed 10% in 72 h treatment (measured by trypan blue exclusion; data not shown). We next investigated whether HIF-1α mediates hypoxia-induced autophagy. The knockdown of HIF-1α with special siRNA significantly abolished the hypoxia-induced expression of LC3-II and Beclin (Figure 3B). These observations suggested that HIF-1α may be required for intermittent hypoxia induced autophagy.

### The intermittent hypoxia induced conversation of non stem cells into stem-like cells is mediated by HIF-1α and autophagy

**Figure 4:**
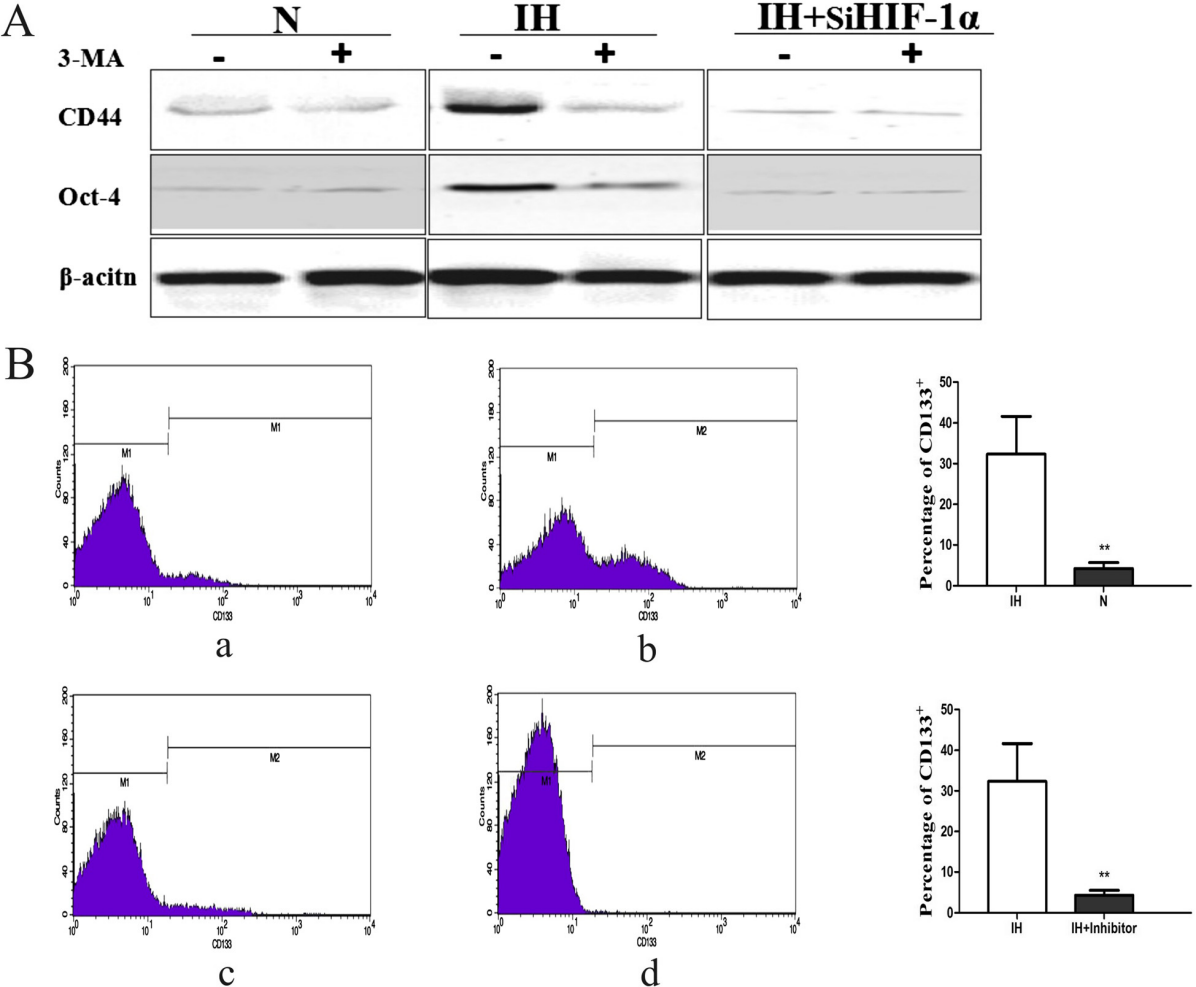
**Blockade HIF-1α and autophagy reduced convertion of non-stem cells into stem-like cells. (A)** Western blot analyses for CD44 and Oct-4 protein levels during intermittent hypoxia (IH) or normoxia (N) with or without HIF-1α siRNA and 3-MA. Data were normalized to β-actin levels. **(B)** The percentage of CD133^+^ subpopulation was reduced under intermittent hypoxia condition with HIF-1α siRNA and 3-MA by flow cytometry. (a) Non-stem pancreatic cancer cells cultured under normoxia without HIF-1α siRNA and 3-MA; (b) Non-stem pancreatic cancer cells cultured under intermittent hypoxia condition without HIF-1α siRNA and 3-MA; (c) Non-stem pancreatic cancer cells cultured under intermittent hypoxia condition with HIF-1α siRNA and 3-MA (IH + Inhibitor); (d) IgG-PE antibody was used as a control. The experiment was repeated 3 times and the data were expressed as mean ± standard deviation. The difference between these three groups was significant, **P < 0.01.

Next, we wondered whether HIF-1α induced autophagy plays an essential role in conversion of non-stem cells into stem-like cells. In intermittent hypoxia knockdown of HIF-1α expression in non-stem pancreatic cancer cells by siRNA with or without 3-methyl adenine (3-MA), the inhibitor of autophagy, it was easily found that the cell morphology changed a spread fibroblast-like into an epithelial-like appearance. Inhibition of HIF-1α and autophagy in intermittent hypoxia significantly reduced Oct-4 and CD44 levels (Figure 4A), while c-MYC did not changed (data not shown). By FACS analysis, compared to the cells under intermittent hypoxia without HIF-1α siRNA and 3-MA group (32.6 ± 5.10%), the proportion of CD133^+^ cells was much smaller in intermittent hypoxia with HIF-1α siRNA and 3-MA group (4.25 ± 0.47%, a 10-fold decrease in CD133 positive population) and in normoxia condition group (4.25 ± 0.36%, a 8-fold decrease in CD133 positive population; Figure 4B). These results suggest that HIF-1α mediated autophagy may be critical for intermittent hypoxia induced conversation of non-stem cancer cells into stem-like cancer cells in Panc-1.

## Discussion

Accumulating data support the concept that the capability of the tumor to grow and propagate is dependent on a small subset of cells within the tumor, termed cancer stem cells. CSCs are responsible for tumor formation, progression, drug resistance, metastasis, and recurrence [17-20]. More and more studies have shown that CSCs can be enriched in cell populations obtained from solid tumors of diverse origins, such as pancreas, breast and brain.

Although the existence of the cancer stem cells in various tumour types had confirmed, the mechanisms regulating their generation and maintenance are still under debate. In particular, there is a clear need for a more detailed characterization of the microenvironmental cues that control the tumour stem cell phenotype. Cancer stem cells are believed to rely on their special microenvironments termed niches to sustain the population. These niches (vascular proliferations, hypoxia/necrosis) represent the hallmarks of malignant tumors [21,22]. Pancreatic cancer is characterized by intratumoral hypovascularity. High levels of hypoxia in pancreatic cancers can be detected intraoperatively with an oxygen microelectrode [23]. More and more evidences confirmed that environmental signals from hypoxic and vascular niches were crucially involved in tumour stem cell maintenance [24-27]. Intermittent hypoxia, which is characterized by cyclic periods of hypoxia and re-oxygenation, occurs in tumor cells that are dependent on tumor blood vessels having intermittent perfusion fluctuations in blood flow. With the aim to more closely mimic solid tumors in *vivo*, we selected a sequence of hypoxic and normoxic intervals of 12 h, namely intermittent hypoxia in this study. One of the open questions is whether hypoxia merely support the self-renewal of CSCs or can also control stem cell plasticity and reprogram the non-stem cancer cells population into CSCs. Recently, several researchers reported that differentiated cancer cells can convert to stem-like cells to maintain equilibrium. In view of our results in *vitro* that the fraction of CD133^+^ cells is found to be enlarged, it is possible that mature CD133^-^ cells can reacquire stem cell properties under intermittent hypoxia. Our results support that non-stem cancer cells could be converted into stem-like cells in the pancreatic cancer cells under intermittent hypoxia.

Tumour hypoxia activates a complex set of cellular responses that are differentially mediated by members of the HIF family. Recent reports have identified new molecular programs by which HIF may regulate cellular differentiation and self renewal, identifying critical stem cell regulators such as Oct4, c-MYC as direct or indirect HIF targets [28-31]. HIF-1α which plays a key role in cancer progression by regulating a number of processes is the best characterized among the transcriptional regulators under hypoxia. In this study, the ablation of HIF-1α with siRNA decreased cells transformation in intermittent hypoxia. Our data supported an additional function of HIF-1α in controlling stem cell plasticity and reprogramming the non-stem cancer cells population into stem-like cells. These results are consistent with a recent report using human glioma cell lines.

Autophagy is an evolutionarily conserved mechanism involving the formation of autophagosomes that sequester cytoplasmic macromolecules and organelles before fusion with the lysosomal compartment. Autophagy was detected by three sets of assays: Western blot of LC3-II protein levels that measures autophagy flux, staining with acridine orange to detect AVOs by fluorescent microscopy and flow cytometry and observation and quantification of green fluorescent protein (GFP)-LC3 puncta by fluorescent microscopy that detects autophagosomes [32-35]. In this study, we used the first assay that measured the protein levels of LC3-II and Beclin1. LC3-II plays an important role in the autophagosome formation. Beclin1 suggests an important role of this protein in cell survival and death relying on the cellular context. Until recently, it remains controversial as to whether autophagy acts primarily as a cell survival or cell death pathway. During hypoxia, autophagy facilitates the removal of ROS-altered mitochondria, reduces oxidative stress and promotes cell survival, but it could also be considered as a pro-tumor mechanism. The present study demonstrated that in the early stagy of intermittent hypoxia autophagy did not only as a survival mechanism under hypoxic conditions but also give a hand in converting the non-stem cancer cells population into stem-like cells. In addition, our results confirm that HIF-1α is a key mechanism in the activation of hypoxia-induced autophagy. But the exact mechanism need further investigation. Since pancreatic carcinoma has a pronounced hypoxic tumour microenvironment and the levels of hypoxia and autophagy are both independent factors, it is of greatly interest in clinical.

## Conclusion

Our results clearly uncover one of the crucial phenomenons of conversation between non cancer stem cells and cancer stem cells in hypoxia and show how HIF-1α and autophagy impact this process in Panc-1. In fact, the other pancreatic cell line (BxPC-3) was also used in this study. Similar results were obtained (data not shown). These results provide an important framework for the development of novel and promised therapeutic strategy that not only more effective and specific anti-CSC therapies but also including the alteration of components of the tumor niche. It may serve as a critical therapeutic paradigm to investigate for the treatment of cancer with the ultimate hope of bringing long-term clinical benefits to the patients.

## Abbreviations

CSCs: Cancer stem cells; HIF-1α: Hypoxia-inducible factor-1α; PCR: Polymerase chain reaction; CD133: Cluster of differentiation 133; CD44: Cluster of differentiation 44; 3-MA: 3-methyl adenine; OCT-4: Octamer-binding transcription factor-4.

## Competing interests

The authors declare no conflict of interests.

## Authors’ contributions

DQ and GB are corresponding author and organized the study. HT, YF and ZZ analyzed data and performed experiments. ZL, LR and MY drafted the manuscript. YP, FF, MY and YY coordinated the study, participated in its design. All authors read and approved the final manuscript.

## References

[B1] Siegel R Naishadham D Jemal A Cancer statistics, 2012 CA Cancer J Clin 2012 62 1 10 29 10.3322/caac.20138 22237781

[B2] Lowery MA O’Reilly EM Pancreatic adenocarcinoma: new approaches to a challenging malignancy Oncology (Williston Park, NY) 2010 24 14 1339 1342 21294480

[B3] Belli C Cereda S Anand S Reni M Neoadjuvant therapy in resectable pancreatic cancer: a critical review Cancer Treat Rev 2013 39 5 518 524 10.1016/j.ctrv.2012.09.008 23122322

[B4] Li C Lee CJ Simeone DM Identification of human pancreatic cancer stem cells Methods Mol Biol (Clifton, NJ 2009 568 161 173 10.1007/978-1-59745-280-9_10 19582426

[B5] Chan KS Volkmer JP Weissman I Cancer stem cells in bladder cancer: a revisited and evolving concept Curr Opin Urol 2010 20 5 393 397 10.1097/MOU.0b013e32833cc9df 20657288 PMC2997612

[B6] Reynolds BA Vescovi AL Brain cancer stem cells: think twice before going flat Cell Stem Cell 2009 5 5 466 467 author reply 468–469 10.1016/j.stem.2009.10.017 19896437

[B7] Al-Hajj M Wicha MS Benito-Hernandez A Morrison SJ Clarke MF Prospective identification of tumorigenic breast cancer cells Proc Natl Acad Sci U S A 2003 100 7 3983 3988 10.1073/pnas.0530291100 12629218 PMC153034

[B8] Singh SK Hawkins C Clarke ID Squire JA Bayani J Hide T Henkelman RM Cusimano MD Dirks PB Identification of human brain tumour initiating cells Nature 2004 432 7015 396 401 10.1038/nature03128 15549107

[B9] Boman BM Wicha MS Cancer stem cells: a step toward the cure J Clin Oncol 2008 26 17 2795 2799 10.1200/JCO.2008.17.7436 18539956

[B10] Clarke MF Dick JE Dirks PB Eaves CJ Jamieson CH Jones DL Visvader J Weissman IL Wahl GM Cancer stem cells–perspectives on current status and future directions: AACR Workshop on cancer stem cells Cancer Res 2006 66 19 9339 9344 10.1158/0008-5472.CAN-06-3126 16990346

[B11] Burness ML Sipkins DA The stem cell niche in health and malignancy Semin Cancer Biol 2010 20 2 107 115 10.1016/j.semcancer.2010.05.006 20510363

[B12] Filatova A Acker T Garvalov BK The cancer stem cell niche(s): the crosstalk between glioma stem cells and their microenvironment Biochim Biophys Acta 2013 1830 2 2496 2508 10.1016/j.bbagen.2012.10.008 23079585

[B13] Calabrese C Poppleton H Kocak M Hogg TL Fuller C Hamner B Oh EY Gaber MW Finklestein D Allen M A perivascular niche for brain tumor stem cells Cancer Cell 2007 11 1 69 82 10.1016/j.ccr.2006.11.020 17222791

[B14] Iliopoulos D Hirsch HA Wang G Struhl K Inducible formation of breast cancer stem cells and their dynamic equilibrium with non-stem cancer cells via IL6 secretion Proc Natl Acad Sci U S A 2011 108 4 1397 1402 10.1073/pnas.1018898108 21220315 PMC3029760

[B15] Hjelmeland AB Wu Q Heddleston JM Choudhary GS MacSwords J Lathia JD McLendon R Lindner D Sloan A Rich JN Acidic stress promotes a glioma stem cell phenotype Cell Death Differ 2011 18 5 829 840 10.1038/cdd.2010.150 21127501 PMC3095828

[B16] Mathieu J Zhang Z Zhou W Wang AJ Heddleston JM Pinna CM Hubaud A Stadler B Choi M Bar M HIF induces human embryonic stem cell markers in cancer cells Cancer Res 2011 71 13 4640 4652 10.1158/0008-5472.CAN-10-3320 21712410 PMC3129496

[B17] Gopalan A Yu W Sanders BG Kline K Eliminating drug resistant breast cancer stem-like cells with combination of simvastatin and gamma-tocotrienol Cancer Lett 2013 328 2 285 296 10.1016/j.canlet.2012.10.003 23063651

[B18] Xu L Cancer stem cell in the progression and therapy of pancreatic cancer Front Biosci (Landmark edition) 2013 18 795 802 10.2741/4143 23747847

[B19] Ribatti D Cancer stem cells and tumor angiogenesis Cancer Lett 2012 321 1 13 17 10.1016/j.canlet.2012.02.024 22388173

[B20] Hermann PC Huber SL Herrler T Aicher A Ellwart JW Guba M Bruns CJ Heeschen C Distinct populations of cancer stem cells determine tumor growth and metastatic activity in human pancreatic cancer Cell Stem Cell 2007 1 3 313 323 10.1016/j.stem.2007.06.002 18371365

[B21] Kingsley LA Fournier PG Chirgwin JM Guise TA Molecular biology of bone metastasis Mol Cancer Ther 2007 6 10 2609 2617 10.1158/1535-7163.MCT-07-0234 17938257

[B22] Denny WA Hypoxia-activated prodrugs in cancer therapy: progress to the clinic Future Oncol 2010 6 3 419 428 10.2217/fon.10.1 20222798

[B23] Koong AC Mehta VK Le QT Fisher GA Terris DJ Brown JM Bastidas AJ Vierra M Pancreatic tumors show high levels of hypoxia Int J Radiat Oncol Biol Phys 2000 48 4 919 922 10.1016/S0360-3016(00)00803-8 11072146

[B24] Soeda A Park M Lee D Mintz A Androutsellis-Theotokis A McKay RD Engh J Iwama T Kunisada T Kassam AB Hypoxia promotes expansion of the CD133-positive glioma stem cells through activation of HIF-1αlpha Oncogene 2009 28 45 3949 3959 10.1038/onc.2009.252 19718046

[B25] Hashimoto O Shimizu K Semba S Chiba S Ku Y Yokozaki H Hori Y Hypoxia induces tumor aggressiveness and the expansion of CD133-positive cells in a hypoxia-inducible factor-1alpha-dependent manner in pancreatic cancer cells Pathobiology 2011 78 4 181 192 10.1159/000325538 21778785

[B26] Kopp HG Avecilla ST Hooper AT Rafii S The bone marrow vascular niche: home of HSC differentiation and mobilization Physiology (Bethesda) 2005 20 349 356 10.1152/physiol.00025.2005 16174874

[B27] Butler JM Kobayashi H Rafii S Instructive role of the vascular niche in promoting tumour growth and tissue repair by angiocrine factors Nat Rev Cancer 2010 10 2 138 146 10.1038/nrc2791 20094048 PMC2944775

[B28] Liang D Ma Y Liu J Trope CG Holm R Nesland JM Suo Z The hypoxic microenvironment upgrades stem-like properties of ovarian cancer cells BMC Cancer 2012 12 201 10.1186/1471-2407-12-201 22642602 PMC3407800

[B29] Higgins LH Withers HG Garbens A Love HD Magnoni L Hayward SW Moyes CD Hypoxia and the metabolic phenotype of prostate cancer cells Biochim Biophys Acta 2009 1787 12 1433 1443 10.1016/j.bbabio.2009.06.003 19524545

[B30] Keith B Johnson RS Simon MC HIF1alpha and HIF2alpha: sibling rivalry in hypoxic tumour growth and progression Nat Rev Cancer 2011 12 1 9 22 22169972 10.1038/nrc3183 PMC3401912

[B31] Semenza GL Hypoxia-inducible factors in physiology and medicine Cell 2012 148 3 399 408 10.1016/j.cell.2012.01.021 22304911 PMC3437543

[B32] Jin S White E Role of autophagy in cancer: management of metabolic stress Autophagy 2007 3 1 28 31 16969128 10.4161/auto.3269 PMC2770734

[B33] Mizushima N Levine B Cuervo AM Klionsky DJ Autophagy fights disease through cellular self-digestion Nature 2008 451 7182 1069 1075 10.1038/nature06639 18305538 PMC2670399

[B34] Chaachouay H Ohneseit P Toulany M Kehlbach R Multhoff G Rodemann HP Autophagy contributes to resistance of tumor cells to ionizing radiation Radiother Oncol 2011 99 3 287 292 10.1016/j.radonc.2011.06.002 21722986

[B35] Li L Chen Y Gibson SB Starvation-induced autophagy is regulated by mitochondrial reactive oxygen species leading to AMPK activation Cell Signal 2013 25 1 50 65 10.1016/j.cellsig.2012.09.020 23000343

